# Pragmatic competence in people with dual diagnosis: down syndrome and autism spectrum disorder

**DOI:** 10.1186/s40359-023-01508-5

**Published:** 2024-02-15

**Authors:** Sara Cortés Escudero, Esther Moraleda Sepúlveda

**Affiliations:** 1 Department of Psychology, Faculty of Health Sciences, University of Castilla-La-Mancha, Avda Real Fábrica de la Seda s/n, 45600 Talavera de la Reina, Spain; 2 Department of Psychology, Faculty of Pychology, University Complutense. Campus de Somosaguas, 28223 Pozuelo de Alarcón. Madrid, Spain

**Keywords:** Autism spectrum disorder (ASD), Down Syndrome (DS), Dual diagnosis, Pragmatic skills, Communication

## Abstract

**Background:**

Pragmatics is an area that can be affected in a wide variety of disorders. In this sense, Syndromic Autism is defined as a disorder in which a causal link is established between an associated syndrome and Autism Spectrum Disorder (ASD). Likewise, Down Syndrome (DS) is one of the main genetically based syndromes in which ASD is described as one of its possible manifestations. In this direction, people with DS are described as social beings whereas in ASD there seems to be a specific alteration of this domain.

**Methods:**

In this study, pragmatic performance was analysed in a sample of 72 participants, where comparisons were made between the scores obtained by children with ASD (n = 24), with DS (n = 24) and with DS + ASD (n = 24).

**Results:**

The Social Communication Questionnaire (SCQ), the Block Objective and Criterial Language Battery (BLOC-SR) and the Neuropsychology subtest (NEPSY-II) aimed at Theory of Mind (ToM) identified significant differences between the groups. However, two-to-two comparisons reported no significant differences between DS and DS + ASD.

**Conclusions:**

Although several studies report differences between the three proposed groups, our data seem to suggest that ASD symptomatology in DS is associated with Intellectual Developmental Disorder (IDD). However, the lack of solid scientific evidence regarding comorbid diagnosis makes further research along these lines indispensable.

**Trial registration:**

This study was approved by the Ethics Committee for Social Research at UCLM with reference CEIS-704,511-L8M4.

## Background

Autism Spectrum Disorder (ASD) is a Neurodevelopmental Disorder characterized by persistent deficits in communication and social interaction [1], as well as restricted and repetitive patterns of behaviors and interests [2], without these deficits being better explained by the presence of an Intellectual Development Disorder (IDD) [3]. In Spain, it is estimated that 15 out of every 1000 school-age children exhibit symptoms compatible with ASD [4]. In this regard, according to data from the National Institute of Statistics (2020), it could be said that the exponential growth in ASD diagnoses over the last three decades, going from an estimated prevalence of 1.1 case per 10,000 inhabitants in 1999 to 3.3 cases per 10,000 inhabitants currently [5], is due to changes in diagnostic criteria [6].

Given all of this, it is not surprising that the diagnosis of ASD is considered a challenging process. However, if we add an underlying genetic syndrome to the mix, the detection becomes even more complex [7]. These cases not classified in diagnostic manuals are known as syndromic autism or “double syndromes,” with an estimated prevalence of 20% [8]. Specifically, authors like Artigas-Pallares, Gabau-Vila & Guitart-Feliubadaló (2005b) reported that Down Syndrome (DS) is one of the main genetically based syndromes in which ASD is described as one of its possible manifestations [9]. In this context, scientific literature has found high prevalence rates for the presence of this comorbidity, ranging from 18 to 38% [10].

In this regard, DS is one of the most common conditions within IDD, commonly known as Intellectual Disability (ID) [11–13]. DS is understood as a congenital syndrome resulting from the presence of an additional chromosome in pair 21 [14, 15], and it is characterized by atypical individual development [16, 17], generalized delays in developmental milestones, personal/social domain [18], adaptive functioning [19, 20] and linguistic development [21].

Based on the aforementioned background and considering various studies confirming the connection between ASD and ID [22], some research has reported increased severity of prototypical ASD symptoms in individuals with genetic syndromes as their Intellectual Quotient (IQ) decreases [23]. However, some studies have described reduced sensitivity and specificity of ASD diagnostic tools, such as the Autism Diagnostic Observation Schedule– 2 (ADOS-2) [24] or the Autism Diagnostic Interview– Revised (ADI-R) [25], when used in individuals with genetic syndromes [26]. From this perspective, analyses by Marlborough et al. (2021) and Thurm et al. (2019) examined the performance of ID in the characteristic behavioral patterns of ASD in genetic disorders [27, 28] such as DS and Fragile X Syndrome (FXS) [29, 30]. As a result, it was concluded that the level of ID did not justify an increased prevalence of clinical ASD characteristics in DS and FXS. It is notable that most studies focused on analyzing the clinical phenotype of the DS + ASD population establish comparisons predominantly with the DS population [10, 26, 29–34]. These comparisons seem to have been based on the assumption that individuals with a comorbid diagnosis, in addition to more severe cognitive, communicative, and behavioral deficits, would also have a specific and distinctive phenotype in relation to ASD. For instance, concerning the cognitive aspect, studies emphasizing the impact on the population with a DS diagnosis and those with a comorbid diagnosis confirm that the latter have lower IQ [10, 34] and limited comprehensive and expressive language skills [35], among others. Therefore, several studies have determined that those with the comorbid diagnosis of DS + ASD exhibit greater impairment in the language component compared to ASD or DS alone [36].

Hence, while research on DS + ASD is ongoing, the early identification of this dual diagnosis is complex due to the overlap of behavioral signs in these disorders [37–39]. Nonetheless, studies have identified two patterns of ASD onset in DS [40, 41]. In the first scenario, research such as that by Hahn, Hamrick, Kelleher & Roberts (2020) or Hepburn, Philofsky, Fidler, et Rogers (2008) showed that ASD symptoms in children with DS diagnosed with ASD emerge early in development, with subjects from both studies demonstrating deficiencies in communication and social skills [42, 43]. Various analyses have corroborated that children with ID and ASD are characterized by inadequate performance in the domains of socialization and communication [44–46]. Regarding the second scenario, Castillo et al. (2008) found a plateau in developmental milestones related to language acquisition and use, as well as social skills, where the regression pattern in those with DS + ASD is similar to those with ASD only [47].

For these reasons, we can assert that these investigations primarily emphasize the importance of pragmatic skills and Theory of Mind (ToM) in the DS + ASD population, identifying significant deficits in areas of social and emotional reciprocity [48], clear limitations in communication skills compared to individuals with DS only, and limited or absent meaningful symbolic communication [49].

## Methods

### Aim

The aim of this present study is to analyze the pragmatic skills of the population with the comorbid diagnosis of DS + ASD to determine the characteristics in this area compared to the population with DS or ASD as a sole diagnosis. The main hypothesis of the study is based on the fact that the pragmatic profile of people with dual diagnosis will be more similar to the population with ASD compared to the population with DS.

### Participants

The selection of participants was established based on the following inclusion criteria: (1) Having a diagnosis of DS, ASD, or DS + ASD conducted by a medical specialist or clinical psychologist, (2) Having a chronological age between 8 and 16 years, and (3) Having Spanish as their native language. On the contrary, the exclusion criteria were: (1) Individuals with a medical diagnosis other than DS, ASD, or DS + ASD, (2) Subjects under 8 years of age, or conversely, over 16 years of age, and (3) Participants whose native language is not Spanish. This range wase selected because it is worth noting that the minimum age set is 8 years, as even though the presence of ASD symptoms in DS manifests in early stages, ensuring a certain level of pragmatic development is necessary and we consider that at the age of 16 adolescence has already ended. Additionally, due to the frequency of studies focusing on the DS + ASD population without a truly diagnosed ASD group, we decided to ensure that all participants in that group had a diagnosis of ASD beyond a screening tool. All participants in the DS + ASD group had a clinical diagnosis conducted by a clinical psychologist based on the results from the ADI-R and ADOS-2 assessments.

Therefore, the study comprises minor users from various Spanish entities and associations. Thus, the research involves 72 participants divided into 3 groups. The first group consists of 24 subjects with DS (13 males and 11 females) with a mean age of 11.6 (SD = 2.9). The second group is composed of 24 individuals with ASD (16 males and 8 females), with a mean age of 11.5 (SD = 2.11). The third and final group is formed by 24 children and adolescents with DS + ASD (17 males and 7 females), with a mean age of 11.1 (SD = 2.21). Furthermore, due to the potential influence of participants’ intellectual capacity on pragmatic components, the level of intelligence was assessed using the NEPSY-II neuropsychological assessment battery [50], revealing no significant differences among the groups. The DS participants had an average IQ of 51.8 (SD = 2.61); the group of children and adolescents with ASD had an average IQ of 52.5 (SD = 3.16), and the group of participants with comorbid diagnosis had an average IQ of 49.3 (SD = 3.09).

### Instruments

The choice of the instruments was based on the fact that they are currently the questionnaires that are most adaptable for people with special needs. For data collection, Form B of the Social Communication Questionnaire (SCQ) [51] was employed. This screening method assesses communicative and social capacities in children aged 4 and above. The questionnaire comprises 40 questions to be answered affirmatively or negatively by the child’s parents or caregivers. The total score is calculated by summing the number of described behaviors marked as “yes.” It is essential to note that the SCQ should never be considered a diagnostic tool but rather as a filter that guides us on whether a more comprehensive evaluation is needed. If a cut-off score exceeding 15 is obtained, the possibility of ASD is considered, warranting further evaluation. To quantify the obtained criteria, the pragmatic module of the Objective and Criterial Language Battery (BLOC-SR) [52] was administered. This standardized test is intended for users aged 5 to 14, evaluating language use in communicative acts through 23 items related to 4 sub-scenes, originating from a specific scene involving a visit to the veterinarian. The interpretation of scores in this section is more intricate than in others, as if the response is literal, a score of 1 point is immediately assigned; otherwise, the examiner must indicate approval or disapproval of the implied content based on whether the response employs the intended pragmatic category. Finally, to assess the prevalence of mentalistic resources, a subtest from the NEPSY-II [50] called “Theory of Mind” (ToM) was employed. This test comprises two specific tasks: verbal and contextual. In the former, based on a specific description or scenario, questions are posed to determine the ability to attribute beliefs or behaviors to a third party. In the latter, there are 21 items that assess 1st and 2nd order task resolution, comprehension of mentalistic verbs, idiomatic phrases, and inferential stories, imitation, integration of information into a coherent whole, and interpretation of facial expressions based on defined conditions. This subtest evaluates recognition of others’ emotions based on a series of images depicting specific social situations.

### Procedure

Initially, contact was established with the Down Syndrome Federation of Castilla - La Mancha and various centers and private clinics in Madrid, Valladolid, Palencia, and Toledo. Information for participation in the project was provided, explaining the potential scope of the study and addressing any queries that may arise. In this regard, an initial meeting was crucial, during which a gathering with patients, caregivers (parents or guardians), and professionals involved in therapeutic intervention took place to ensure understanding of the study and explain the roles of participants and the significance of their involvement. This step empowered them to make decisions freely and knowingly, thereby guaranteeing their informed and voluntary participation. Once the groups agreed, completed, signed, and submitted the informed consent form, a date was scheduled for the administration of assessment tests. The consent had to be expressed by the parent/guardian as the legal representative of the minor or dependent individual. Subsequently, individual sessions of 30 min were arranged for each family, during which the speech therapist conducted the subject’s assessment while parents completed the SCQ. This study was approved by the Ethics Committee for Social Research at University of Castilla-La Mancha with reference CEIS-704,511-L8M4.

### Data analysis

Firstly, an exploratory analysis was conducted by examining measures of central tendency (mean and median) and their measures of dispersion. Subsequently, the normality of distribution was assessed through tests of normality, shape statistics (skewness and kurtosis), and normal Q-Q plots with and without trend. The normality of the sample was assessed using the Kolmogorov–Smirnov test, which indicated that the data were non-parametric. Specifically, the Shapiro-Wilk test was employed given that the sample size for each group did not exceed 50 participants. All variables were found to be non-parametric (*p* < 0.5). Additionally, in order to determine significant differences among the variables, the Kruskal-Wallis analysis of variance test was used, as the variables are quantitative and do not follow a normal distribution.

## Results

Firstly, the data reveal that the group comprising children and adolescents with ASD achieves a higher mean score than the groups consisting of individuals with DS and DS + ASD in the SCQ questionnaire. Conversely, they attain a lower mean score compared to the other two groups in the BLOC assessment (Table 1).

**Table 1:** Mean scores in the different utilized tests

	SCQ	BLOC	ToM
DS Group	16.5 (4.43)	36.3 (17.7)	2.54 (1.18)
ASD Group	22.6 (4.97)	16.5 (15.9)	1.08 (0.88)
DS + ASD Group	16.4 (4.36)	44.6 (23.2)	2.21 (0.83)

Standard deviation in parenthesis

Continuing, the analysis of results concerning the SCQ was undertaken. The results have revealed significant differences (X2(2) = 20.021; *p* < 0.001) in the total communicative competence level among children with ASD, children with DS + ASD, and children with DS. Specifically, it was observed that all three groups exhibited symptomatology consistent with ASD by surpassing the questionnaire’s cutoff point of 15 points. However, the group consisting of individuals with ASD obtained a higher mean score compared to the other two groups. Pairwise comparisons demonstrated that while significant differences in pragmatic competence level were observed between the DS and ASD groups (W(2) = 5.34; *p* < 0.001) and between the ASD and DS + ASD groups (W(2) = -5.46; *p* < 0.01), such differences were absent when comparing the DS and DS + ASD groups (W(2) = -0.04; *p* > 0.05).

However, when considering the grouping of SCQ items into the three core areas of ASD diagnosis, no significant differences were found between groups: social interaction (H(2) = 3.52; *p* > 0.05), communication difficulties (H(2) = 0.20; *p* > 0.05), and restricted, repetitive, and stereotyped behavior (H(2) = 039; *p* > 0.05).

Moving on to the BLOC results, they once again demonstrated statistically significant differences (H(2) = 20.40; *p* < 0.001) in pragmatic competence level among children with ASD, children with DS, and children with DS + ASD. In this regard, pairwise comparisons indicated significant differences in pragmatic competence between DS and ASD (W = -5.06; *p* < 0.001) and between ASD and DS + ASD (W = 5.68; *p* < 0.001). Conversely, no significant differences were found when comparing groups of children with DS and DS + ASD (W = 1.79; *p* > 0.05).

Finally, the results regarding the Theory of Mind (ToM) subtest indicated statistically significant differences among groups (H (2) = 21.1; *p* < 0.001), with the ASD group obtaining the lowest scores. Pairwise comparisons revealed significant differences between the groups comprising individuals with DS and ASD (W = -5.65; *p* < 0.001) and between those with ASD and DS + ASD (W = 5.47; *p* < 0.001). Conversely, no differences were found between groups composed of individuals with DS and DS + ASD (W = -1.41; *p* > 0.05).

## Discussion

The objective of the present investigation focused on analyzing whether there were differences in pragmatic skills between the population presenting isolated DS or ASD compared to the comorbid presentation of DS + ASD. This inquiry was prompted by the contentious debate within the scientific community regarding whether the pragmatic alterations commonly associated with ASD observed in the DS population are secondary to the DS or instead linked to comorbidity between both disorders. The results have revealed that the group of children and adolescents with a comorbid diagnosis of DS + ASD exhibited similar characteristics to the DS group and differed in skills from the group with ASD, contrasting with other studies [53, 54]. For example, Channell et al. (2015) reported that individuals with DS showing ASD symptomatology displayed a more pronounced developmental delay, characterized by frequent disruptions in social communication and socioemotional reciprocity compared to those with isolated DS [55]. Similarly, Dressler et al. (2011) evaluated peer relationships [56] using the Vineland Adaptive Behavior Scale (VABS) [57] and found greater impairment in DS + ASD, in contrast to results obtained using the Childhood Autism Rating Scale (CARS) [58], which suggested favorable scores in DS + ASD as opposed to those with ASD.

This disparity in the profile of pragmatic skills reflected in the scientific literature could stem from the fact that the majority of scientific publications employ surveys or interviews with parents aimed at diagnosing ASD, attempting to define symptomatology consistent with the disorder and its severity [35]. From this perspective, the study by Warner et al. (2017), based on the application of the SCQ [51], compared 183 children with ASD to 189 children with DS who scored above 15 on this screening test. The results demonstrated significant differences between both groups in SCQ scores [59], in line with our findings. However, the authors discovered that distinctions primarily occurred in the domain of reciprocal social interaction, unlike our study. This can be attributed to differing perspectives on DS, distinguishing between those who perceive these individuals as sociable beings [60] and those who demonstrate pragmatic difficulties [61].

It appears, therefore, that the findings regarding pragmatic development in these populations suggest that while pragmatic aspects pose challenges in ASD [62], DS [61], and DS + ASD [63], their pragmatic competence differs when compared to each other. Thus, the DS + ASD group [36] seems to exhibit relative patterns of proficiency in this competence when compared to DS. This aligns with prior research by Dressler et al. (2011), which found that, based on the CARS [58], individuals with DS + ASD exhibit greater similarity to those with DS, significantly differing from those with ASD, especially in the imitation skills and interactive pragmatics present in personal relationships [56]. For this reason, it is expected that the DS population tends to be affable and sociable [64], thus not exhibiting deficits in their social and pragmatic skills [60].

On the other hand, focusing on the comparison between DS and ASD, we find that the pragmatic level of DS is conceived as a tool for communication and social interaction, as previously shown in Ferrario’s research [65]. Conversely, ASD presents a pronounced deficit in this component. In this regard, Adamson et al. (2009), comparing 23 children with ASD and 29 children with DS to 56 typically developing children, found that the ASD group exhibited lower intersubjectivity due to more pronounced alterations compared to the DS group [66]. This corroborates our findings, with the ASD group scoring lower than the DS and DS + ASD groups in the pragmatics section of the BLOC.

Furthermore, Godfrey et al. (2019), using the ADI-R [25], assessed individuals with ASD, DS, and DS + ASD, observing that, based on parental criteria, the ASD group, in contrast to the DS + ASD group, displayed a higher index of prototypical ASD-associated behaviors. Likewise, the DS + ASD group exhibited symptoms more similar to ASD than DS, contrary to our results [63].

The obtained data also indicated differences in Theory of Mind (ToM) skills performance among groups of children and adolescents with DS and ASD, as well as between groups of children and adolescents with ASD and DS + ASD. Along these lines, the findings of this study can be interpreted in line with previous research that demonstrated better performance by the DS population compared to the ASD population [67]. Although no literature has been found that directly compares ToM task performance in the DS + ASD population, it would have been expected that they would perform worse than the other two groups, given the existing literature suggesting a more severe deficit profile. However, our data did not reveal significant differences between groups consisting of individuals with DS and DS + ASD, which implies that the ASD-compatible symptomatology detected in the DS population could arise due to the DS itself [68]. Regarding the relationship between Theory of Mind and pragmatic abilities, some authors such as [69], found that in people with ASD, group with “Lower ToM abilities” was characterized by more severe ASD symptoms and poorer pragmatic skills, in terms of inappropriate communicative beginnings and deficits in coherence and interpretation of language depending on the context, among other indicators. This group also showed significantly less mastery of daily living skills and poorer adaptive skills than the “Higher ToM abilities” profile, which showed less widespread impairment. This relationship has also been documented in the case of Down Syndrome [70]. Similarly, the absence of differences in ToM skills between the DS and DS + ASD groups may be influencing the results obtained in the BLOC. This test requires the use of these skills to successfully complete it, as participants need to put themselves in the position of another to respond as they should in a given social situation.

Finally, it is important to highlight that early diagnosis is necessary to implement an effective therapy tailored to the child’s needs at a critical time in development [71]. Appropriate early treatment can reduce children’s symptoms and improve their overall development and quality of life, allowing them to gain social skills and the ability to act better in social situations, in order to achieve more autonomy in later life [72]. It is for this reason that it must be taken into account that a precise and correct diagnosis in these populations will serve as a starting point to propose the most appropriate intervention possible.

## Conclusions

Therefore, this research confirms significant differences between the clinical DS and ASD groups, as well as between the ASD and DS + ASD groups, in both the scores obtained in the SCQ and the BLOC, as well as in the ToM task. However, no differences were found between the DS and DS + ASD groups in any of the tests. Thus, our data suggest that there are no differences in the pragmatic performance of the DS + ASD population compared to the isolated presentation of ASD. Nevertheless, the lack of robust scientific evidence regarding comorbid diagnosis, coupled with the fact that the available studies suggest a clustering of more severe ASD symptomatology in the DS + ASD population, makes further investigation in this direction essential.

## Data Availability

All data from this study are available upon request to the corresponding author.

## Abbreviations

ADOS-2: Autism Diagnostic Observation Schedule– 2
ADI-R: Autism Diagnostic Interview– Revised
ASD: Autism Spectrum Disorder
BLOC S-R: Block Objective and Criterial Language Battery
DS: Down Syndrome
FXS: Fragile X Syndrome
ID: Intellectual Disability
IDD: Intellectual Developmental Disorder
IQ: Intellectual Quotient
SCQ: Social Communication Questionnaire
ToM: Theory of Mind
